# Fatty Acid Esters of Hydroxy Fatty Acids (FAHFAs) Are Associated With Diet, BMI, and Age

**DOI:** 10.3389/fnut.2021.691401

**Published:** 2021-07-12

**Authors:** Teresa Kellerer, Karin Kleigrewe, Beate Brandl, Thomas Hofmann, Hans Hauner, Thomas Skurk

**Affiliations:** ^1^Else Kröner-Fresenius-Center of Nutritional Medicine, TUM School of Life Sciences, Technical University of Munich, Freising, Germany; ^2^Bavarian Center for Biomolecular Mass Spectrometry (BayBioMS), TUM School of Life Sciences, Technical University of Munich, Freising, Germany; ^3^Core Facility Human Studies, ZIEL Institute for Food and Health, Technical University of Munich, Freising, Germany; ^4^Chair of Food Chemistry and Molecular Sensory Science, TUM School of Life Sciences, Technical University of Munich, Freising, Germany; ^5^Institute of Nutritional Medicine, TUM School of Medicine, Technical University of Munich, Munich, Germany

**Keywords:** body weight, diet, diabetes mellitus, bariatric surgery, obesity, fatty acid esters of hydroxy fatty acids

## Abstract

**Background:** Fatty acid esters of hydroxy fatty acids (FAHFAs) are a group of fatty acids with potential anti-inflammatory and anti-diabetic effects. The blood levels of FAHFAs and their regulation in humans have hardly been studied.

**Objective:** We aimed to investigate serum FAHFA levels in well-characterized human cohorts, to evaluate associations with age, sex, BMI, weight loss, diabetic status, and diet.

**Methods:** We analyzed levels of stearic-acid-9-hydroxy-stearic-acid (9-SAHSA), oleic-acid-9-hydroxy-stearic-acid (9-OAHSA) and palmitic-acid-9-hydroxy-palmitic-acid (9-PAHPA) as well as different palmitic acid-hydroxy-stearic-acids (PAHSAs) by HPLC-MS/MS with the use of an internal standard in various cohorts: A cohort of different age groups (18–25y; 40–65y; 75–85y; *Σn* = 60); severely obese patients undergoing bariatric surgery and non-obese controls (*Σn* = 36); obese patients with and without diabetes (*Σn* = 20); vegetarians/vegans (*n* = 10) and omnivores (*n* = 9); and young men before and after acute overfeeding with saturated fatty acids (SFA) (*n* = 15).

**Results:** Omnivores had substantially higher FAHFA levels than vegetarians/vegans [median (25th percentile; 75th percentile) tFAHFAs = 12.82 (7.57; 14.86) vs. 5.86 (5.10; 6.71) nmol/L; *P* < 0.05]. Dietary overfeeding by supplementation of SFAs caused a significant increase within 1 week [median tFAHFAs = 4.31 (3.31; 5.27) vs. 6.96 (6.50; 7.76) nmol/L; *P* < 0.001]. Moreover, obese patients had lower FAHFA levels than non-obese controls [median tFAHFAs = 3.24 (2.80; 4.30) vs. 5.22 (4.18; 7.46) nmol/L; *P* < 0.01] and surgery-induced weight loss increased 9-OAHSA level while other FAHFAs were not affected. Furthermore, significant differences in some FAHFA levels were found between adolescents and adults or elderly, while no differences between sexes and between diabetic and non-diabetic individuals were detected.

**Conclusions:** FAHFA serum levels are strongly affected by high SFA intake and reduced in severe obesity. Age also may influence FAHFA levels, whereas there was no detectable relation with sex and diabetic status. The physiological role of FAHFAs in humans remains to be better elucidated.

**Trial Registration:** All studies referring to these analyses were registered in the German Clinical Trial Register (https://www.drks.de/drks_web/) with the numbers DRKS00009008, DRKS00010133, DRKS00006211, and DRKS00009797.

## Introduction

Besides their principal function as an energy source, lipids are essential as components of cell membranes and as precursors of signaling molecules. As such, some lipids serve as bioactive mediators regulating various biological effects after binding to specific receptors. Dysregulation of lipid mediators' endogenous levels has been linked to inflammation, atherosclerosis, and various other diseases (1, 2).

In 2014, a group of bioactive lipids, the fatty acid esters of hydroxy fatty acids (FAHFAs), has been reported (3). These molecules consist of a fatty acid coupled to a hydroxy fatty acid by an ester bond. Whereas the discovery of 16 family members of FAHFAs is described in the original publication, more lipid combinations have been shown since then (4–8), and, theoretically, every possible combination of a fatty acid with a hydroxy fatty acid is conceivable.

FAHFAs have been detected in different foods, either from plants and from animal sources (3, 7, 9). To date, there is little knowledge concerning the fate of dietary FAHFAs upon oral intake. They may be enzymatically digested, as FAHFA hydrolases have been identified (10–12). It is assumed that the levels of FAHFAs in serum and adipose tissue are mainly derived from the endogenous synthesis in adipocytes (3, 4). Yet, data on the impact of diet on FAHFA serum levels are still missing. Moreover, the endogenous synthesis pathway is not yet explored, but, seems to be connected to adipose tissue lipogenesis as a knockout of ChREBP, which regulates adipose tissue lipogenesis, reduces PAHSA levels (3). Besides, the synthesis of FAHFAs seems to be stereoselective, supporting the assumption that synthesis *in vivo* is enzymatically mediated by an unidentified acyl transferase (13).

The recent discovery of FAHFAs has evoked growing interest, as studies have shown that these bioactive lipids might have potent anti-inflammatory properties (3, 4, 14–16). Moreover, they are considered to mediate anti-diabetic effects by stimulating insulin secretion and increasing insulin-stimulated glucose uptake into the adipocyte (3, 15). These effects have been shown particularly for palmitic-acid-hydroxy-stearic-acid (PAHSA), which represents one of the most predominant groups of FAHFAs in humans and mice. However, a recent study could not support the anti-diabetic effects of PAHSAs in mice (17).

Due to their potentially favorable metabolic effects, FAHFAs as natural-occurring, bioactive lipids may have some potential for new treatment strategies in the context of metabolic and inflammatory diseases (14). Research about FAHFAs is only at the outset and, by now, only very few studies have addressed human FAHFA levels under different physiological and pathophysiological conditions. It is known from the original publication, that serum levels of PAHSA are positively correlated with insulin sensitivity in humans (3). Additionally, a further recent publications reported on specific FAHFAs or groups of FAHFAs being affected by the glycemic state (18) and body mass index (BMI) of individuals (6, 18).

Despite their potential therapeutically implication for e.g., metabolic and inflammatory diseases only very few human data are available. Therefore, the present study aimed to analyze FAHFA levels in different human cohorts to assess their typical physiological abundance and to evaluate the influence of age, sex, BMI, weight loss, and glycemic state on the regulation of those levels. We also put a special focus on different types of diet (animal-based, vegetarian/vegan), which clearly seem to influence abundance of FAHFA levels in different cohorts.

## Materials and Methods

### Reagents

FAHFA standards [5-PAHSA, 9-PAHSA, ^13^C_4_-9-PAHSA, 10-PAHSA, 12-PAHSA, 13-PAHSA, stearic-acid-9-hydroxy-stearic-acid (9-SAHSA), oleic-acid-9-hydroxy-stearic-acid (9-OAHSA) and palmitic-acid-9-hydroxy-palmitic-acid (9-PAHPA)] were purchased from Biomol (Hamburg, Germany). Sodium chloride was ordered from Sigma-Aldrich (Steinheim, Germany). All solvents of chromatographic grade (methanol, chloroform, hexane and ethyl acetate) and sodium citrate tribasic dihydrate were from Merck (Darmstadt, Germany).

### Human Samples and Cohorts

If not otherwise stated, we collected serum samples from clinical studies conducted in the same study unit (Human Study Center, EKFZ, Freising, Germany). Written informed content was obtained from all participants before their inclusion in the respective study. All procedures were performed according to the principles of the Declaration of Helsinki.

To investigate a potential association with age and sex, three different cross-sectional age cohorts were used which were recruited and characterized in the course of the *enable* cluster at the study center in Freising, Germany. The *enable* study (DRKS0009797) investigated anthropometrics, body composition, energy metabolism, and dietary habits in defined life phases. For this study, we included adolescents (18–25 years), middle-aged adults (40–65 years), and older people (75–85 years) (19). All study participants were without any severe acute diseases. From each age cohort, we randomly selected 10 females and 10 males for FAHFA-measurements. Adolescents and adults were all average weight (BMI 18.5–25 kg/m^2^), while the BMI range for seniors was extended up to 30 kg/m^2^ due to the natural increase in BMI with higher age. The group of older people also showed slightly but significantly increased fasting glucose levels and HOMA-IR compared to the younger groups (Supplementary Table 1), but levels were still in the normal range (20, 21), except for the HOMA-IR of one adult and one older person (5.16 and 3.09, respectively).

The cohort of obese patients undergoing bariatric surgery and non-obese controls originated from a recent study on gut permeability (22) (DRKS00009008). The *n* number in this publication and the original publication differs according to the in- or exclusion of patients with medications that might influence the respective analysis. In this study, severely obese patients (BMI ≥ 40 kg/m^2^) were investigated before as well as 6 months after bariatric surgery (laparoscopic sleeve gastrectomy) and in comparison to non-obese (BMI < 30 kg/m^2^) control participants (Supplementary Table 2).

For the comparison of patients with and without diabetes, samples were provided from the adult *enable* cohort and the cross-sectional German Obesity Biomaterial Bank (GOBB). In the *enable* cohort, volunteers with elevated fasting glucose levels (≥ 126 mg/dL) and/or a 2 h blood glucose value of ≥200 mg/dL in the oral glucose tolerance test (OGTT) were characterized as newly diagnosed patients with diabetes (*n* = 8). They were compared with the non-diabetic adult population of the *enable* cohort (*n* = 20). The diabetic group had significantly higher fasting glucose and insulin levels and showed higher body weight and BMI (Supplementary Table 3).

Participants from the obese GOBB cohort were classified as “obese diabetic” with a BMI ≥ 40 kg/m^2^ and HbA1c ≥ 6.5% and “obese non-diabetic” with a BMI ≥ 40 kg/m^2^ and HbA1c <5.7%. None of the patients did take insulin or oral antidiabetic medication. There were 5 females and 5 males in each group, and these groups did not differ in age, body weight, BMI and C-reactive protein (CRP) levels (Supplementary Table 4).

The influence of dietary factors was studied in vegetarians/vegans (*n* = 10) and omnivores (*n* = 5) from the JPI-project FOODBALL (23) (DRKS00010133) as well as omnivores from the study on gut permeability (*n* = 4) of which two subjects were undergoing laparoscopic abdominal surgery. Omnivores were a cross-sectional group of healthy, lean (BMI < 25 kg/m^2^) volunteers that habitually followed a normal mixed diet including meat and fish (three females, six males) and vegetarians/vegans were a group of vegetarian or vegan volunteers practicing a vegan diet for at least 7 days (seven females, three males). Groups differed in age but were similar in body weight, BMI, and fasting glucose levels (Supplementary Table 5).

A group undergoing a 1-week overfeeding intervention originated from a recently published study cohort of 15 lean (BMI < 27 kg/m^2^), healthy, young men (24) (DRKS00006211). We performed overfeeding by providing a daily surplus of 1,000 kcal above the participants' calculated energy demand based on their measured resting metabolic rate. The additional energy was added mainly as saturated fatty acids from whipping cream. The participants had a median weight gain of 1.2 (0.6; 1.4) kg of body weight during the period of overfeeding. Fasting blood glucose levels and levels of NEFAs decreased while fasting insulin and CRP levels were unaltered (Supplementary Table 6).

### Anthropometric Measurements

Measurements were made without shoes and outdoor clothing such as jackets and coats. The measurements were corrected by one kg. In *enable*, FOODBALL and the over-nutrition study, measurements were performed in underwear without correction. Height was determined using a stadiometer and body weight with a calibrated standard scale (Seca, Hamburg, Germany).

### Blood Samples and Biochemical Analyses

Blood samples were drawn in the fasting state. In patients with severe obesity, blood samples were taken during bariatric surgery in cooperating hospitals. Blood was drawn into a serum monovette (S-Monovette^®^ 7.5 mL Z-Gel, 01.1602, Sarstedt, Nümbrecht, Germany) and clot for 30 min at room temperature and was subsequently centrifuged at 2,500 g for 10 min. The serum was aliquoted and stored at −80°C until analysis. Non-esterified fatty acids (NEFAs) were measured in plasma using a commercial test kit (Wako Chemicals GmbH, Neuss, Germany). Blood glucose was determined either by SynLab (Munich, Germany) or in frozen blood plasma with the Hemocue system (Hemocue^®^ Glucose 201+, Radiometer GmbH, Krefeld, Germany). Blood glucose of the patients with obesity was recorded from the lab reports of the respective clinic. Insulin in plasma was either quantified by Synlab (Munich, Germany) or by commercially available ELISA kits (DRG Diagnostics, Marburg, Germany; DAKO, Glostrup, Denmark). HOMA-IR of corresponding samples was calculated according to Matthews (25): HOMA-IR = insulin [μU/mL] × glucose (mmol/L)/22.5.

### FAHFA Extraction

FAHFA extraction from human serum samples was performed according to an adapted version of the protocol by Zhang and colleagues (26). Blood specimens from the different groups of one cohort were randomly assigned to FAHFA extraction batches, and one blank sample (H_2_O) was included in each batch. 0.9 mL of serum sample was used, and samples were spiked with an isotope-labeled internal standard (10 μL of 0.1 μg/mL ^13^C_4_-9-PAHSA in methanol). Extraction of lipids was performed with 2 mL of citric acid buffer (100 mM sodium citrate tribasic dihydrate, 1 M sodium chloride, pH 3.6), 3 mL of methanol, and 6 mL of chloroform and a subsequent centrifugation at 2,200 g for 10 min at 4°C.

For FAHFA enrichment, SPE columns (HyperSep silica SPE column, 500 mg bed weight, 6 mL column volume; Thermo Scientific, Darmstadt, Germany) were used and washed with 8 mL of 95:5 (vol/vol) hexane:ethyl acetate and 8 mL of ethyl acetate and conditioned with 15 mL of hexane. Further steps were performed as in (26). For a detailed description of LC-MS/MS-analysis, please see Supplementary Data. The structure of the FAHFAs analyzed can be found in Supplemental Figure 1. The expression tPAHSAs corresponds to the total sum of PAHSAs analyzed (9-, 10-, 12/13-PAHSA), and the expression tFAHFAs (total FAHFA) corresponds to the total sum of FAHFAs analyzed (PAHSAs, 9-OAHSA, 9-SAHSA, 9-PAHPA).

### Statistical Analysis

All data in the tables are presented as median (25th percentile; 75th percentile). Data are graphically presented in boxplots that are defined as follows: the bottom and the top of the box are defined by the 25th and 75th percentile and the center by the median. The whiskers reach until the lowest data point still within 1.5 IQR (interquartile range) of the lower quartile, and the highest data point still within 1.5 IQR of the upper quartile. Outliers are indicated by dots. For FAHFA analysis, non-parametric tests were used due to the small sample size and non-Gaussian distribution of most of the FAHFA data. The Wilcoxon matched-pairs test was used to compare the same group at two different time points. Mann-Whitney test was used to compare two independent groups. Differences in the three different age groups were detected by the Kruskal-Wallis test and, in case of significant difference, the Dunn's post-test. Correlation analysis was performed by Spearman correlation. *P* < 0.05 was regarded as statistically significant. Statistical analyses and graphical outputs were performed with GraphPad Prism 5 (GraphPad Software, San Diego California, USA) and the R programming environment (R studio, Version 3.3.2).

## Results

### FAHFA Concentrations Depending on Age and Sex

**Figure 1:**
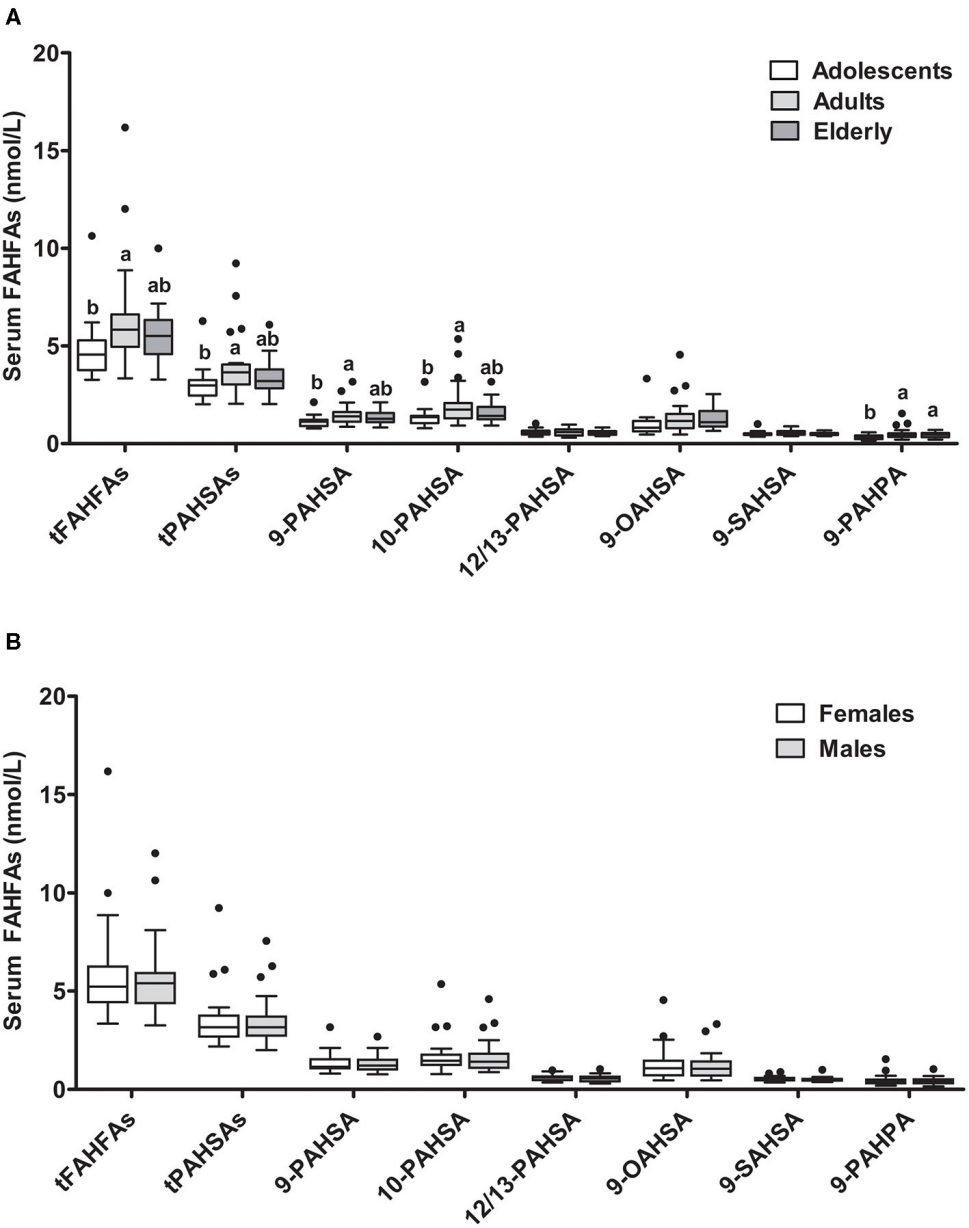
FAHFA concentrations in different age groups and sexes. Serum FAHFA levels were measured in the *enable* cohort according to age **(A)** and sex **(B)**. Adolescents, 18–25 years; Adults, 40–65 years; Elderly, 75–85 years. Kruskal-Wallis test with Dunn's posttest was used to compare the age groups. Mann-Whitney test was used to compare females and males. Box plots describe the median and the 25th percentile and 75th percentile. *n* = 20 per age group and *n* = 30 per sex. Labeled medians without a common superscript letter differ, *P* < 0.05. t, total (sum of FAHFAs/PAHSAs measured); FAHFA, fatty acid esters of hydroxy fatty acid; OAHSA, oleic acid-hydroxy stearic acid; PAHPA, palmitic acid-hydroxy palmitic acid; PAHSA, palmitic acid-hydroxy stearic acid; SAHSA, stearic acid-hydroxy stearic acid.

FAHFA concentrations in different age groups of the *enable* cluster revealed that levels of 9-PAHPA, 9-PAHSA, and 10-PAHSA, as well as tPAHSAs and tFAHFAs, (t = total, sum of measured fatty acid esters) were higher in the adult group compared with the adolescents. In the elderly, levels were not significantly elevated than those of adolescents, except for 9-PAHPA (Figure 1A). When subjects were divided according to sex, no difference between the female and male groups was detectable (Figure 1B).

We also investigated possible correlations between FAHFA levels and anthropometric and metabolic parameters. When all three age groups were analyzed together, there were no significant correlations between levels of tFAHFAs or tPAHSAs and anthropometric or metabolic variables. A separate adult group analysis showed significantly negative correlations of BMI with tFAHFAs (rho = −0.67, *P* = 0.001) and tPAHSAs (rho = −0.50, *P* = 0.023), respectively.

### BMI and FAHFA Concentrations

**Figure 2:**
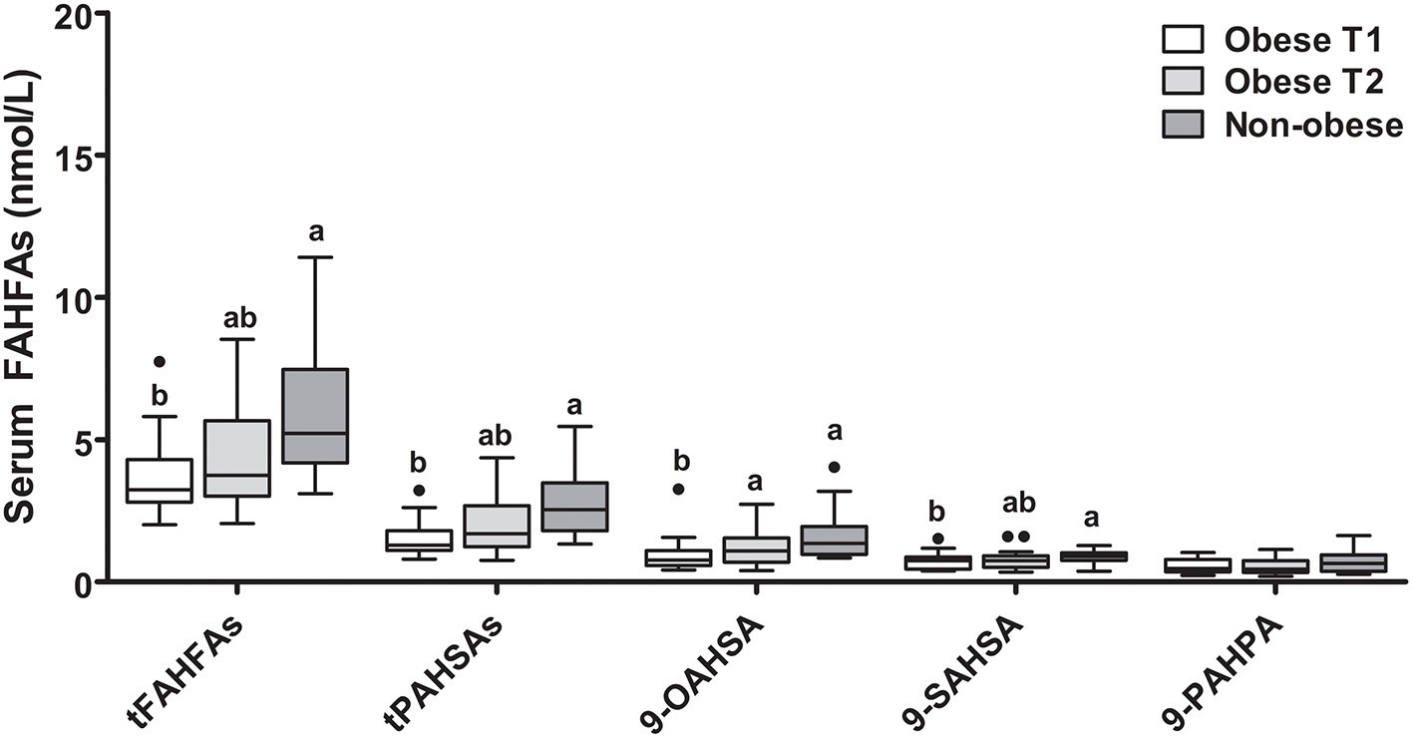
FAHFA concentrations in morbidly obese individuals before and 6 months after laparoscopic sleeve gastrectomy compared to non-obese volunteers. Wilcoxon matched-pairs test was used to compare obese patient before and after surgery (Obese T1 and Obese T2) and Mann-Whitney test to compare obese patients with non-obese patients. Box plots describe the median and the 25th percentile and 75th percentile. *n* = 18 per group. Labeled medians without a common superscript letter differ, *P* < 0.05. t, total (sum of FAHFAs/PAHSAs measured); FAHFA, fatty acid ester of hydroxy fatty acid; OAHSA, oleic acid-hydroxy stearic acid; PAHPA, palmitic acid-hydroxy palmitic acid; PAHSA, palmitic acid-hydroxy stearic acid; SAHSA, stearic acid-hydroxy stearic acid.

FAHFA levels in a group of 18 morbidly obese subjects (BMI ≥ 40 kg/m^2^) before (T1) and 6 months after bariatric surgery (laparoscopic sleeve gastrectomy) (T2) compared to 18 age- and sex-matched non-obese controls (BMI < 30 kg/m^2^) showed, that, except for 9-PAHPA, the levels of all other FAHFAs depicted were significantly different between non-obese and obese participants (T1) with higher levels in the non-obese group (Figure 2). At 6 months after surgery (T2), the median weight loss of patients with obesity was 39.5 (30.7; 44.7) kg, and the median BMI drop was 13.6 (10.8; 15.6) kg/m^2^. Together with weight loss, the patients' metabolic parameters improved, measured by a decrease in fasting glucose and insulin, NEFA levels, and CRP concentration (Supplementary Table 2). The levels of 9-OAHSA were increased at 6 months after surgery. Other FAHFAs were not significantly altered (Figure 2).

We also investigated the role of anthropometric, metabolic, and dietary factors on tPAHSA and tFAHFA levels. When obese patients before surgery and non-obese controls were analyzed together, negative correlations were seen for tPAHSAs with levels of NEFAs (rho = −0.65; *P* < 0.001), CRP (rho = −0.55; *P* < 0.001), and insulin (rho = −0.37; *P* = 0.029) as well as for tFAHFAs with NEFAs (rho = −0.62; *P* < 0.001) and CRP (rho = −0.46; *P* = 0.006). To exclude the influence of BMI, the non-obese and obese groups were also analyzed separately, and no significant correlations were seen between tFAHFAs or tPAHSAs and CRP, insulin, or NEFAs.

### FAHFA Concentrations According to the Metabolic Status

**Figure 3:**
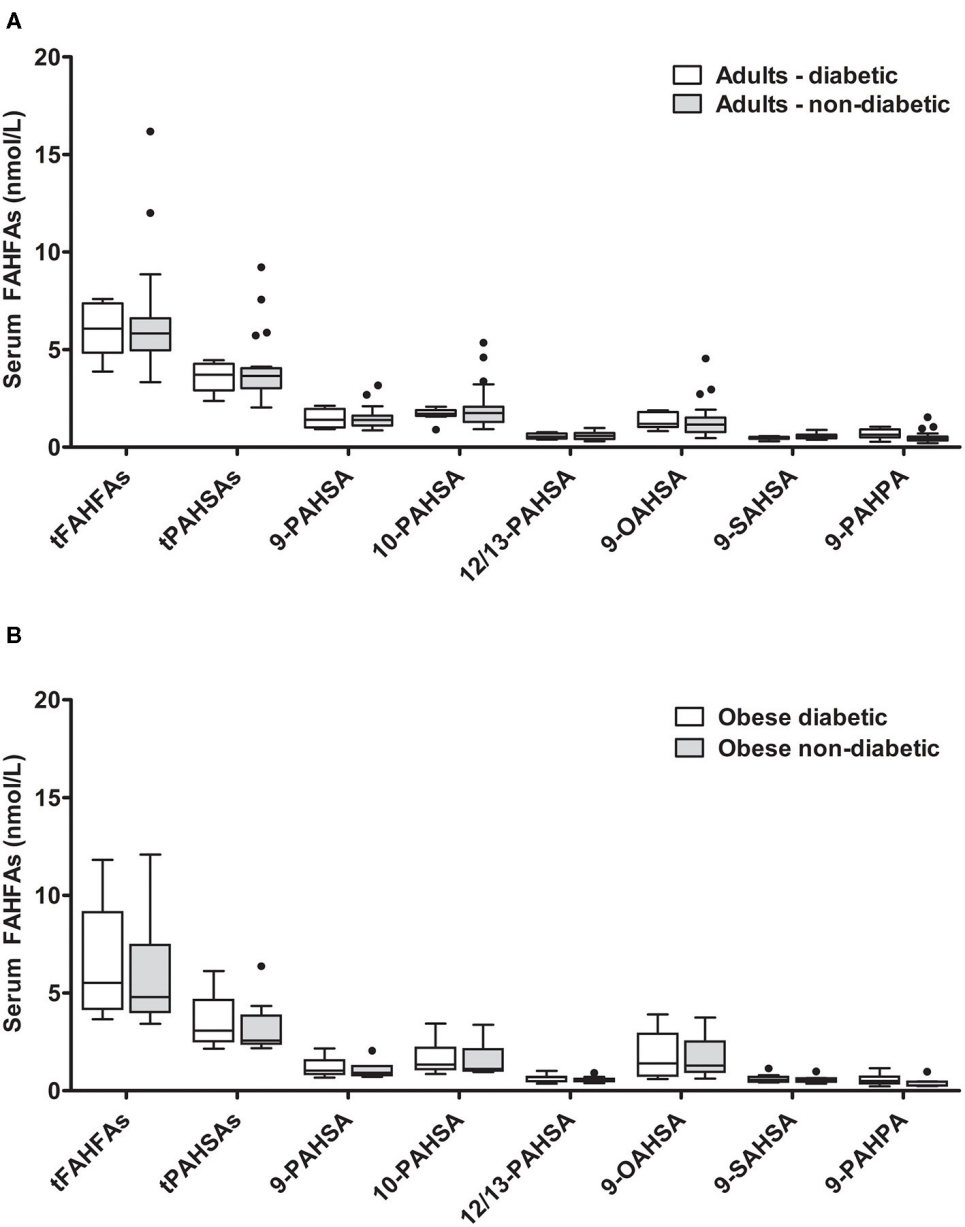
FAHFA concentrations in diabetic and non-diabetic individuals. **(A)** FAHFA concentration was measured in diabetic and non-diabetic individuals from the *enable* cluster. Mann-Whitney test was used to compare the groups. *n* = 8/20 (diabetic/non-diabetic). **(B)** FAHFA concentration was measured in obese diabetic and obese non-diabetic individuals from the GOBB cohort. Mann-Whitney test was used to compare the groups. Box plots describe the median and the 25th percentile and 75th percentile. *n* = 10 per group. t, total (sum of FAHFAs/PAHSAs measured); FAHFA, fatty acid ester of hydroxy fatty acid; OAHSA, oleic acid-hydroxy stearic acid; PAHPA, palmitic acid-hydroxy palmitic acid; PAHSA, palmitic acid-hydroxy stearic acid; SAHSA, stearic acid-hydroxy stearic acid.

It was previously reported that PAHSA levels are reduced in insulin-resistant subjects (3). Patients with diabetes from our adult *enable* cohort (*n* = 8) were therefore compared with the non-diabetic adult population (*n* = 20). The diabetic group had significantly higher fasting glucose and insulin levels and presented higher body weight and BMI (Supplementary Table 3). The levels of all different FAHFAs measured did not differ between diabetic and non-diabetic adult individuals (Figure 3A). No significant correlations were seen between tFAHFA or tPAHSA levels and anthropometric or metabolic parameters when patients with and without diabetes were analyzed together.

Additionally, we compared diabetic and non-diabetic participants from a cohort of obese patients (GOBB). Obese patients with diabetes had significantly higher blood glucose levels and higher HbA1c (Supplementary Table 4). No significant differences were found for the FAHFAs analyzed between obese diabetic and obese non-diabetic patients (Figure 3B).

### Vegetarianism and Overfeeding Determine FAHFA Concentrations

**Figure 4:**
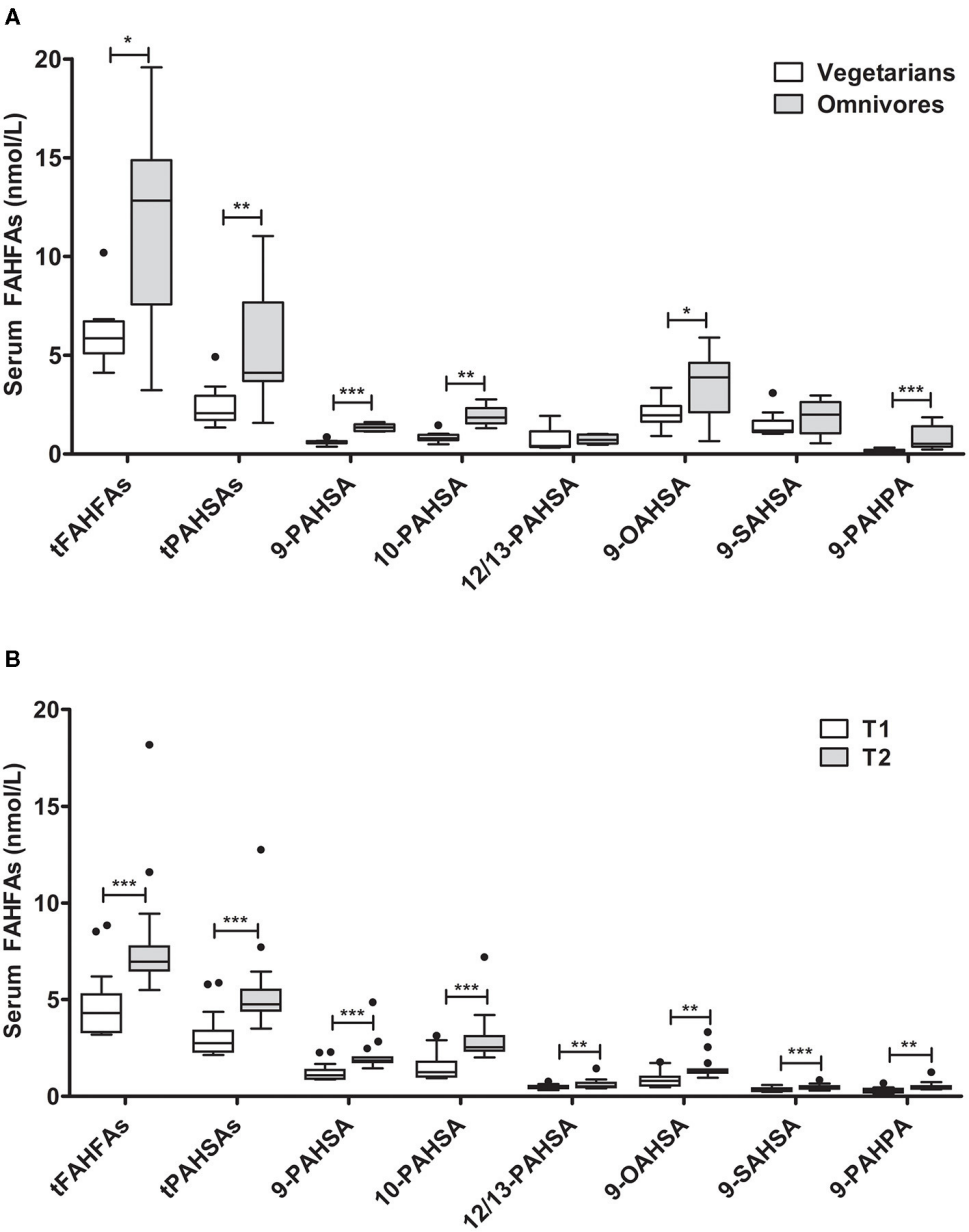
FAHFA concentrations in vegetarians/vegans compared to omnivores and in lean young men before and after acute overfeeding. **(A)** FAHFA concentration in individuals following a vegetarian or vegan diet (vegetarians) in comparison to individuals under a normal diet (omnivores). Mann-Whitney test was used to compare the groups. *n* = 10/9 (vegetarians/omnivores), except for 9-, 10- and 12/13-PAHSA: *n* = 10/5. **(B)** FAHFA concentration in young, healthy males undergoing short-term overfeeding. Wilcoxon matched-pairs test was used to compare participants before (T1) and after (T2) intervention. Box plots describe the median and the 25th percentile and 75th percentile. *n* = 15. *P < 0.05; **P < 0.01; ***P < 0.001. t, total (sum of FAHFAs/PAHSAs measured); FAHFA, fatty acid ester of hydroxy fatty acid; OAHSA, oleic acid-hydroxy stearic acid; PAHPA, palmitic acid-hydroxy palmitic acid; PAHSA, palmitic acid-hydroxy stearic acid; SAHSA, stearic acid-hydroxy stearic acid.

We further investigated the association of dietary habits with systemic FAHFA levels. Vegetarians were slightly younger than omnivores (22 vs. 28 years) but were similar in body weight, BMI, and fasting glucose levels (Supplementary Table 5). We detected significantly lower levels of all FAHFAs analyzed in the vegetarian group than the omnivore group, except for 9-SAHSA and 12/13-PAHSA (Figure 4A), which were similar.

To further explore the impact of diet on FAHFA abundance in serum samples, we analyzed FAHFAs under the condition of overfeeding with 1,000 kcal above the calculated energy demand, mainly as saturated fatty acids from whipping cream for 1 week (24). The participants had a significant median weight gain of 1.2 (0.6; 1.4) kg of body weight during the period of overfeeding. Fasting blood glucose levels and levels of NEFAs decreased during the study period, while fasting insulin and CRP levels were unaltered (Supplementary Table 6). The FAHFA concentrations in serum of participants increased significantly after 1 week of overfeeding (Figure 4B). Spearman correlation analysis revealed no significant association at baseline between anthropometric, metabolic, or dietary parameters and tPAHSA or tFAHFA levels, except for CRP and FAHFAs (rho = −0.56; *P* = 0.0499). We did not find a significant correlation between the increase in tFAHFA or tPAHSA levels and changes in anthropometric, metabolic, or dietary parameters.

## Discussion

The present work aimed to measure serum levels of FAHFAs in different human cohorts and to investigate their associations with BMI, dietary factors, age, sex, and metabolic parameters. Following previously published protocols for FAHFA extraction of human serum samples and LC-MS/MS analysis (3, 26, 27), we could quantitatively analyze 9-OAHSA, 9-SAHSA, 9-PAHPA as well as PAHSAs, namely 9-, 10-, and 12/13-PAHSA. Separation between 12- and 13-PAHSA was not achieved, as already reported by other groups (3, 26, 27), but we quantified both combined. We also detected peaks for 5-PAHSA; however, the MRM transition ratios did not always match the corresponding standard. This was probably due to a previously observed overlay of 5-PAHSA with a ceramide contaminant (26, 27). We, therefore, excluded 5-PAHSA from our analysis. The levels of FAHFAs detected in human serum were in the low nanomolar range and slightly lower, yet comparable to the levels in human serum analyzed by Yore and colleagues (3) and higher than those measured by Zhu et al. (28). This may be due to differences in the analytical procedures and sensitivity of the respective methods. However, although this analytical variability might limit the comparability to the other studies, it does not interfere with the interpretation within our homogenous and well-defined human cohorts.

First, we investigated the association of age and sex with serum FAHFA levels. While sex does not seem to be related to FAHFA levels, we found higher levels of most FAHFAs in adults (40–65y) compared to adolescents (18–25y), but levels were lower again in the oldest cohort (75–85y). This observation is surprising; however, it needs further investigation whether nutrition or the endogenous metabolism is responsible for the finding. Interestingly, a recent study in mice could show significant correlations between age and FAHFA levels at least in visceral adipose tissue (29).

Next, we investigated the effect of BMI on serum levels of different FAHFAs. The group of morbidly obese patients revealed lower FAHFA levels in blood compared to the non-obese control volunteers. Although the pattern seems to shift toward non-obese controls, a significant decrease in body weight at 6 months after bariatric surgery did not significantly alter serum FAHFA levels, except for an increase in 9-OAHSA. Therefore, a more extended period of follow-up would be of interest to clarify if FAHFAs remain reduced or may slowly increase toward the non-obese levels. In this context, it is necessary to mention that blood samples of patients with obesity were taken during surgery, and we, therefore, cannot exclude confounding by the surgical intervention.

A previous study in mice showed that high-fat diet-induced obesity resulted in tissue- and isomer-specific regulation of PAHSAs; while some PAHSAs were downregulated in serum, others were not (3). Similarly, in humans, lower levels of palmitoleic acid-hydroxy palmitic acid (POHPA) have been reported in overweight compared to normal-weight subjects, but no differences were seen for other groups of FAHFAs (18). Another recent study also reported significant differences for some but not all FAHFAs in human plasma between lean and obese/overweight subjects; however, overweight/obese subjects showed increased levels of those FAHFAs compared to lean subjects (6). Another group reported reduced levels of PAHSAs in the breast milk of obese compared to lean mothers (30). Therefore, our own study and previous studies by others indicate that BMI has an impact on FAHFA levels, and this impact seems tissue-specific and isomer-specific. Interestingly, FAHFAs can also be incorporated in triacylglycerols (TGs), and FAHFA-TGs can serve as a depot for FAHFAs in adipocytes, as recently discovered (31). The incorporation of FAHFAs into TGs and the FAHFA release of those TGs may therefore play an important role in serum FAHFA regulation, especially in a state of increased adipose tissue mass.

PAHSAs have been credited with potent anti-diabetic effects. The administration of PAHSAs to mice resulted in improved insulin sensitivity and glucose tolerance (3, 15, 32). These effects were related to enhanced insulin secretion, directly induced at the beta-cell level via G-protein coupled receptor (GPR)-40, as well as by an enhanced GLP-1 secretion in the gut (3, 15, 33). Moreover, insulin-stimulated glucose uptake in the adipocyte was promoted via binding to the GPR-120 (3). Accordingly, reduced PAHSA levels might represent one mechanism by which obesity contributes to insulin resistance. We, therefore, compared FAHFA levels in individuals with and without type 2 diabetes both in the GOBB and in the *enable* cohort. We could not detect any difference in FAHFA levels between diabetic and non-diabetic individuals in these two cohorts. Moreover, no correlations between fasting glucose, fasting insulin or HOMA-IR, and tFAHFAs or tPAHSAs were seen in these groups. In the original publication by Yore and colleagues (3), reduced levels of PAHSAs in insulin-resistant compared to insulin-sensitive subjects have been observed. However, both groups differed in BMI, which may have contributed to this result. Also, we used glucose and clinical diagnosis to classify our study subjects. Of note, another recent study reported even negative correlation of 9-POHSA and 9-OAHSA with blood glucose in healthy subjects (16). An interpretation of all those studies might be complicated by the fact that different experimental set ups were used and confirmatory experimental studies with a similar design over a longer period are needed to clarify the role of FAHFAs in diabetes and insulin resistance.

It is also worth noting that we found significantly lower levels of FAHFAs in subjects eating a plant-based diet (vegetarian/vegan) compared to omnivores, despite the appraisal of plant-based diets as being protective against the development of diabetes (34). Therefore, it seems doubtful to assume that low FAHFA levels are an independent risk factor to develop type 2 diabetes despite the slightly significant difference in age between vegetarians and omnivores. Both study groups can still be classified as “young” (22 and 28 years) compared to our adult cohort (mean age 56 years) and it is therefore unlikely that age accounts for the observed difference. Lower FAHFA levels in vegetarians/vegans might also seem controversy due to the suggested anti-inflammatory nature of a plant based diet (Haghighatdoost, 2017), which could be attributed to the low fat content of the diet, which in turn is in line with our findings in the group of young men undergoing overfeeding for 1 week. Here, a significant increase in FAHFA levels was observed after the intervention compared to baseline, despite unaltered insulin sensitivity measured by the euglycemic hyperinsulinemic clamp technique (24). Lower serum FAHFAs in obesity compared to overfeeding might be related to higher adipose HSL (hormone sensitive lipase)-production (35) in the higher adipose tissue mass in obesity. HSL might cause increased break-down of FAHFA-ester bonds as shown recently (36). It seems therefore related to the long-term development of adipose tissue mass, which is supported by the fact that the basal levels are comparable between the cohort of young man and the non-obese control group for our bariatric patients.

To date, only little is known about the origin of FAHFAs in circulation. FAHFAs have been found in different foods (3, 7, 9), and a recent study could show that subjects ingesting liposomal oat oil rich in LAHLA (linoleic acid esters of hydroxy linoleic acids) had markedly elevated serum LAHLA levels compared to controls (37). Interestingly, animal products, such as beef and egg, seem to contain more FAHFAs compared to plant products (3), which would explain our finding of higher FAHFA levels in omnivores compared to vegetarians. However, several FAHFA hydrolases have been described (10–12), and dietary FAHFAs may thus be enzymatically digested upon oral intake.

Next to dietary uptake, FAHFAs can also be synthesized endogenously in adipose tissue via esterification of hydroxy fatty acids with fatty acids. This has already been shown in mice where dietary supplementation with the non-endogenous hydroxy fatty acid 9-hydroxy heptadecanoic acid (9-HHA) was used to detect the corresponding FAHFA 9-palmitic-acid-hydroxy-heptadecanoic-acid (9-PAHHA) in the blood (3). Furthermore, it was assumed that levels of FAHFAs in serum and adipose tissue mainly reflect endogenous synthesis rather than dietary intake, as isomer distribution in the diet differs from isomer distribution in serum and tissue (3). Moreover, serum FAHFA levels may be regulated via the synthesis and breakdown of FAHFA-TGs in adipocytes (31). Therefore, we assume that the higher concentrations of FAHFAs in the serum of omnivores in comparison to vegetarians/vegans do not necessarily result from higher intakes of FAHFAs with the diet. The fat content and fatty acid composition of an ovo-lacto-vegetarian or vegan diet, however, differs from that of a diet containing fish and meat, as vegetarians/vegans have lower intakes of total fat and saturated fatty acids and a higher n6:n3 fatty acid ratio (38, 39). The differences in amount and type of fatty acids that are taken up and deposited in adipocytes may influence the endogenous synthesis of FAHFAs. For example, vegans have a relatively high intake of linoleic acid (38). Linoleic acid can also be incorporated in FAHFAs, like linoleic acid-hydroxy linoleic acid (LAHLA) (37). It, therefore, can be speculated that levels of other FAHFAs like LAHLAs, which we did not measure in the present study, might be higher in vegans compared to omnivores.

Furthermore, our overeating study results may point to an influence of the amount and type of fatty acid uptake on FAHFA synthesis. The intake of a surplus of 1,000 kcal, mainly containing high amounts of saturated fatty acids, resulted in a marked increase in circulating FAHFAs. These FAHFAs predominantly contain saturated fatty acids (palmitic and stearic acid) and only 9-OAHSA as a monounsaturated fatty acid (oleic acid). We speculate that the levels of FAHFAs containing other unsaturated or poly-unsaturated fatty acids (4) may be differently modulated after the overfeeding period. However, analysis by Spearman rank correlation revealed no significant association for the rise in tFAHFAs or tPAHSAs and changes in parameters, such as total energy intake, total fat intake, and saturated fat intake. Tracer studies are certainly needed to finally investigate the origin of circulating FAHFA with regard to endogenous production or dietary intake. Interestingly, in this study, the high SFA diet resulted in decreased NEFAs which could be explained by specific fat matrix provided (24).

In conclusion, we here described the predominant impact of nutrition to FAHFA levels in serum. The results are underscored in an independent group of well-designed cohort and intervention studies. However, also some phenotypic influence to the FAHFA abundance could be demonstrated. As a novel finding, we could show that acute short-term over-feeding with whipping cream significantly increased FAHFA levels in young adults with no obvious impact on insulin-sensitivity measured by the gold standard of the euglycemic, hyperinsulinemic clamp technique. Moreover, subjects on a plant-based diet had substantially lower levels of FAHFAs compared to omnivores. These data suggest a profound influence of specific dietary habits on FAHFA abundance in serum. Besides, in our cohorts, we observed a varying influence of age on FAHFA levels as well as lower levels in obesity, while sex and diabetic status did not seem to have a major impact on serum FAHFA concentrations.

This manuscript describes an exploratory study with potential limitations. One might be a small sample size combined with high variability in FAHFA levels, potentially reducing the power to detect differences, especially since we were comparing various readouts like age, sex, BMI, weight loss, diabetic status, and diet, each for numerous FAHFAs, which potentially causes false-positive error rates. Moreover, confounding is always an issue in observational studies. We tried to match groups for various factors; however, residual confounding may remain. As we analyzed only a subset of FAHFAs, our results do not represent the whole group of FAHFAs in human serum. Technical improvements should aim for more discriminative FAHFA analysis. The definite strength of the study is the broad overview above a broad spectrum of nutritional influences on FAHFA levels.

## Data Availability Statement

The raw data supporting the conclusions of this article will be made available by the authors, without undue reservation.

## Ethics Statement

The studies involving human participants were reviewed and approved by Ethics Committee of the Faculty of Medicine at the Technical University of Munich. The patients/participants provided their written informed consent to participate in the studies.

## Author Contributions

TS, HH, KK, and TK designed the research and planned the experiments. TS, HH, TK, and BB were responsible for the human studies and provided serum samples. TK performed sample preparation and FAHFA extraction. TK and KK set up the HPLC-MS/MS method, analyzed the samples, and performed the analysis. TH provided necessary analysis equipment and technical support. TK, TS, HH, and KK wrote the manuscript. All authors have read and approved the manuscript.

## Conflict of Interest

The authors declare that the research was conducted in the absence of any commercial or financial relationships that could be construed as a potential conflict of interest.
