# Supplementary material for: Fatty Acid Esters of Hydroxy Fatty Acids (FAHFAs) Are Associated With Diet, BMI, and Age
Source: Front Nutr. 2021 Jul 12;8:691401. doi: 10.3389/fnut.2021.691401 (PMC8310931; doi:10.3389/fnut.2021.691401)
Supplement: Supplementary file 1 [file Table_1.docx]

**Supplemental Table 1: Characteristics of individuals from three different age groups of the *enable* study^1^.**

|  | Median  (25^th^ percentile; 75^th^ percentile) | | |
| --- | --- | --- | --- |
|  | **Adol.** | **Adults** | **Elderly** |
| **n (m/f)** | **20 (10/10)** | **20 (10/10)** | **20 (10/10)** |
| **Age** | 22^c^  (21; 23) | 56^b^  (49; 58) | 77^a^  (75; 80) |
| **Body weight (kg)** | 66.2  (58.9; 74.4) | 66.3  (54.2; 75.0) | 69.2  (61.9; 78.4) |
| **Body mass index (kg/m²)** | 20.4^b^  (19.9; 21.3) | 21.8^b^  (20.6; 23.1) | 24.9^a^  (22.8; 26.9) |
| **Fasting blood glucose (mg/dL)** | 77^b^  (73; 81) | 78^b^  (72; 82) | 87^a^  (81; 89) |
| **Fasting insulin (mg/dL)** | 2.0  (2.0; 3.2) | 2.0  (2.0; 2.9) | 2.9  (2.0, 6.1) |
| **HOMA-IR** | 0.39^b^  (0.37; 0.58) | 0.39^b^  (0.36; 0.58) | 0.62^a^  (0.42; 1.32) |
| **CRP (mg/dL)** | 0.04^b^  (0.02; 0.06) | 0.06^a,b^  (0.04; 0.12) | 0.13^a^  (0.08; 0.19) |
| **Fat intake (g/day)** | 84.51  (70; 96) | 82.62  (78; 93) | 79.33  (61; 108) |
| **SFA intake (g/day)** | 37.64  (31; 45) | 36.18  (32; 43) | 36.14  (27; 44) |

^1^ Adol. = Adolescents: 18-25 years; Adults: 40-65 years; Elderly: 75-85 years. Depending on the distribution of the data Kruskal-Wallis test with Dunn’s post-test or one-way ANOVA and Tukey post-test were used to compare the groups. Values are median (25^th^ percentile; 75^th^ percentile).. Superscript letters a,b,c stand for a significant relation. Labelled medians in a row without a common superscript letter differ significantly, *P*<0.05.

CRP = C-reactive protein, SFA = saturated fatty acid

**Supplemental Table 2: Characteristics of obese patients undergoing bariatric surgery and non-obese controls^1^.**

|  | Median  (25^th^ percentile; 75^th^ percentile) | | |
| --- | --- | --- | --- |
|  | **Obese T1** | **Obese T2** | **Non-obese** |
| **n (m/f)** | **18 (2/16)** | **18 (2/16)** | **18 (2/16)** |
| **Age** | 46  (35; 50) | - | 47  (35; 51) |
| **Body weight (kg)** | 150.5^a^  (133.4; 175.8) | 109.5^b^  (93.9; 136.6) | 63.3^c^  (56.2; 73.4) |
| **Body mass index (kg/m²)** | 52.6^a^  (46.2; 60.4) | 39.3^b^  (31.4; 45.0) | 21.6^c^  (19.6; 23.6) |
| **Fasting blood glucose (mg/dL)** | 95^a^  (82; 104) | 83^b^  (74; 90) | 89^a,b^  (84; 94) |
| **Fasting insulin (mg/dL)** | 38.3^a^  (26.9; 69.2) | 26.0^b^  (16.4; 39.2) | 11.5^c^  (9.5; 18.0) |
| **CRP (mg/dL)** | 0.55^a^  (0.39; 0.99) | 0.30^b^  (0.13; 0.56) | 0.03^c^  (0.02; 0.06) |
| **NEFA (mmol/L)** | 1.01^a^  (0.92; 1.54) | 0.54^b^  (0.35; 0.91) | 0.32^c^  (0.25; 0.41) |
| **Total energy intake (kcal/day)** | 1747^b^  (1407; 2080) | 881^c^  (757; 1060) | 1990^a^  (1793; 2305) |
| **Fat intake (g/day)** | 66^a^  (56; 86) | 37^b^  (30; 40) | 79^a^  (70; 98) |
| **SFA intake (g/day)** | 28.5^a^  (23; 33) | 15.7^b^  (14; 19) | 37.32^c^  (28;45) |

^1^ Depending on the distribution of the data Wilcoxon matched-pairs test or paired t-test was used to compare obese patient before and after surgery (Obese T1 and Obese T2) and Mann-Whitney test or unpaired t-test to compare obese patients with non-obese controls. Values are median (25^th^ percentile; 75^th^ percentile).. Superscript letters a,b,c stand for a significant relation. Labelled medians in a row without a common superscript letter differ significantly, *P*<0.05.

CRP = C-reactive protein; NEFA = non-esterified fatty acid; SFA = saturated fatty acid

**Supplemental Table 3: Characteristics of adult diabetic and non-diabetic individuals of the *enable* cohort^1^.**

|  | Median  (25^th^ percentile; 75^th^ percentile) | | *P*-value |
| --- | --- | --- | --- |
|  | **Diabetic** | **Non-diabetic** | **Diabetic**  **–**  **Non-diabetic** |
| **n (m/f)** | **8 (6/2)** | **20 (10/10)** |  |
| **Age** | 55  (49; 64) | 56  (49; 58) | n.s. |
| **Body weight (kg)** | 98.4  (95.9; 108.9) | 66.3  (54.2; 75.0) | *** |
| **Body mass index (kg/m²)** | 31.9  (30.7, 33.4) | 21.8  (20.6; 23.1) | *** |
| **Fasting blood glucose (mg/dL)** | 118  (93; 126) | 78  (72; 82) | *** |
| **Fasting insulin (mg/dL)** | 10.9  (7.8; 14.7) | 2.0  (2.0; 2.9) | *** |
| **HOMA-IR** | 3.00  (1.90; 4.35) | 0.39  (0.36; 0.58) | *** |
| **CRP (mg/dL)** | 0.32  (0.29; 0.43) | 0.06  (0.04; 0.12) | ** |

^1^ Depending on the distribution of the data Mann-Whitney test or unpaired t-test was used to compare the groups. Values are median (25^th^ percentile; 75^th^ percentile). . Not significant (n.s.) *P*≥0.05; ***P*<0.01, ****P*<0.001.

CRP = C-reactive protein

**Supplemental Table 4: Characteristics of obese diabetic and non-diabetic individuals of the GOBB cohort^1^.**

|  | Median  (25^th^ percentile; 75^th^ percentile) | | *P*-value |
| --- | --- | --- | --- |
|  | **Obese diabetic** | **Obese non-diabetic** | **Diabetic**  **-**  **non-diabetic** |
| **n (m/f)** | **10 (5/5)** | **10 (5/5)** |  |
| **Age** | 44  (36; 47) | 42  (35; 45) | n.s. |
| **Body weight (kg)** | 159.5  (138.8; 171.1) | 145.5  (122.7; 184.4) | n.s. |
| **Body mass index (kg/m²)** | 53.6  (47.3; 57.3) | 46.7  (42.2; 59.0) | n.s. |
| **Fasting blood glucose (mg/dL)** | 124  (96; 223) | 82  (73; 97) | ** |
| **HbA1c (%)** | 7.1  (6.7; 9.1) | 5.2  (5.1; 5.3) | *** |
| **CRP (mg/dL)** | 1.64  (0.89; 3.00) | 1.35  (0.60; 2.80) | n.s. |

^1^ Depending on the distribution of the data Mann-Whitney test or unpaired t-test was used to compare the groups. Values are median (25^th^ percentile; 75^th^ percentile).. Not significant (n.s.) *P*≥0.05; ***P*<0.01, ****P*<0.001.

CRP = C-reactive protein

**Supplemental Table 5: Characteristics of vegetarians and omnivores of the FOODBALL cohort^1^**.

|  | Median  (25^th^ percentile; 75^th^ percentile) | | *P*-value |
| --- | --- | --- | --- |
|  | **Vegetarians** | **Omnivores** | **Vegetarians**  **-**  **Omnivores** |
| **n (m/f)** | 10 (3/7) | 9 (6/3) |  |
| **Age** | 22  (20; 23) | 28  (26; 36) | ** |
| **Body weight (kg)** | 63.8  (56.4; 76.3) | 81.3  (56.8; 84.1) | n.s. |
| **Body mass index (kg/m²)** | 21.6  (19.8; 22.6) | 23.0  (20.4; 24.9) | n.s. |
| **Fasting blood glucose (mg/dL)** | 88  (86; 90) | 92  (89; 100) | n.s. |

^1^ Depending on the distribution of the data Mann-Whitney test or unpaired t-test was used to compare the groups. Values are median (25^th^ percentile; 75^th^ percentile).. Not significant (n.s.) *P*≥0.05; ***P*<0.01.

**Supplemental Table 6: Characteristics of young men undergoing overfeeding^1^.**

|  | Median  (25^th^ percentile; 75^th^ percentile) | | *P*-value |
| --- | --- | --- | --- |
|  | **T1** | **T2** | **T1 – T2** |
| **n (m/f)** | **13 (13/0)** | **13 (13/0)** |  |
| **Age** | 22  (21; 24) | - | - |
| **Body weight (kg)** | 79.6  (66.8; 82.8) | 79.6  (68.1; 84.2) | *** |
| **Body mass index (kg/m²)** | 23.3  (21.3; 24.9) | 23.5  (21.7; 25.2) | *** |
| **Fasting blood glucose (mg/dL)** | 85  (78; 87) | 80  (71; 84) | * |
| **Fasting insulin (mg/dL)** | 4.3  (4.0; 5.0) | 4.5  (3.9; 5.2) | n.s. |
| **HOMA-IR** | 0.9  (0.8; 1.1) | 0.9  (0.8; 1.0) | n.s. |
| **CRP (mg/dL)** | 0.02  (0.01; 0.03) | 0.04  (0.01; 0.04) | n.s. |
| **NEFA (mmol/L)** | 0.37  (0.28; 0.54) | 0.17  (0.12; 0.23) | *** |
| **Total energy intake (kcal/day)** | 2570  (2330; 3490) | 4015  (3910; 4252) | *** |
| **Fat intake (g/day)** | 112  (80; 131) | 214  (209; 227) | *** |
| **SFA intake (g)** | 50  (32; 58) | 114  (112; 117) | *** |

^1^ Depending on the distribution of the data Wilcoxon matched-pairs test or paired t-test was used to compare participants before (T1) and after (T2) intervention. Intervention consisted of a 7 day overfeeding with a surplus of 1000 kcal/day. Values are median (25^th^ percentile; 75^th^ percentile). *n* = 15; with the exception of CRP measurement: . Not significant (n.s.) *P*≥0.05; **P*<0.05, ***P*<0.01, ****P*<0.001.

CRP = C-reactive protein; NEFA = non-esterified fatty acid; SFA = saturated fatty acid

**Supplemental Methods**

**LC – MS/MS Analysis**

The chromatographic conditions are based on the work of Yore et al. (Yore et al., 2014) , Zhang *et al*. (Zhang et al., 2016) and Kolar *et al.* (Kolar et al., 2016). For LC-MS analysis, samples were thawed and reconstituted in 100 µL of methanol. Chromatographic separation was performed with a Nexera UPLC equipped with a DGU-20A5R degassing unit, a LC-30AD pump, a SIL-30AC autosampler unit and a CTO-30A column oven (Shimadzu, Duisburg, Germany). A BEH C18 column (100 x 2.1 mm) with 1.7 µm particle size was used (Waters, Milford, MA, USA). Injection volume was 10 µL and flow rate was 300 µL/min at 40°C. The running buffer used to separate FAHFAs was 93:7 methanol/water with 5 mM ammonium acetate and 0.03% ammonium hydroxide (v/v) under isocratic conditions. For the quantitation of FAHFAs UPLC was coupled to a QTRAP 6500 mass spectrometer with IonDrive Turbo V source and the software Analyst Version 1.6.2. (AB Sciex, Darmstadt). Negative electrospray ionization mode was used. Further MS-settings are summarized in **Supplemental Table 7**.

A calibration curve of a mixture containing all FAHFA standards was used for quantitation. Seven different concentrations of FAHFAs in methanol were used for the calibration curve, ranging from 0 to 100 ng/mL. The FAHFA standards were as follows: 5-PAHSA, 9-PAHSA, 10-PAHSA, 12-PAHSA, 13-PAHSA, 9-SAHSA, 9-OAHSA, and 9-PAHPA. The structure of the FAHFAs analyzed can be found in **Supplemental Figure 1**. The chromatographic separation of FAHFA standards is presented in **Supplemental Figure 2**. For each of these FAHFAs, two multiple reaction monitoring (MRM) transitions were measured. The first one was used as quantifier and a second one as qualifier. ^13^C_4_-PAHSA was used for all FAHFAs as isotopically labeled standard. The MRM transitions and the optimized collision energy and cell exit potential are listed in **Supplemental Table 8**. Only those FAHFAs were quantified, whose retention time and ion ratios of the quantifying and qualifying MRM transition fitted the corresponding standard. The expression tPAHSAs corresponds to the total sum of PAHSAs analyzed (9-, 10-, 12/13-PAHSA) and the expression tFAHFAs corresponds to the total sum of FAHFAs analyzed (PAHSAs, 9-OAHSA, 9-SAHSA, 9-PAHPA).

**Supplemental Table 7: Optimised MS parameters for FAHFA detection.**

| Parameter | Setting |
| --- | --- |
| IonSpray Voltage | -4,500 V |
| Source temperature | 450 °C |
| Curtain gas | 35 psi |
| Ion source gas 1 | 55 psi |
| Ion source gas 2 | 65 psi |
| Collison gas | nitrogen |
| Collision activated dissociation (CAD) | -2 |
| Declustering potential (DP) | -40 V |
| Entrance potential (EP) | -10 V |

**Supplemental Table 8: FAHFA specific MS parameters^1^.**

| FAHFA Compound |  | Q_1_ (*m/z*) | Q_3_ (*m/z*) | Dwell Time (msec) | Collision Energy (CE) | Cell Exit Potential (CXP) |
| --- | --- | --- | --- | --- | --- | --- |
| ^13^C_4_-PAHSA |  | 541.5 | 259.1 | 100 | -48 | -15 |
| PAHSA | Quantifier [M-H]^-^  Qualifier [M-H]^-^ | 537.5  537.5 | 255.1  281.2 | 100  100 | -48  -38 | -10  -1 |
| OAHSA | Quantifier [M-H]^-^  Qualifier [M-H]^-^ | 563.3  563.3 | 281.0  299.2 | 100  100 | -38  -38 | -31  -15 |
| SAHSA | Quantifier [M-H]^-^  Qualifier [M-H]^-^ | 565.3  565.3 | 282.8  281.1 | 100  100 | -42  -38 | -27  -29 |
| PAHPA | Quantifier [M-H]^-^  Qualifier [M-H]^-^ | 509.3  509.3 | 255.1  253.1 | 100  100 | -36  -34 | -27  -25 |

^1^ FAHFA = fatty acid ester of hydroxy fatty acid; OAHSA = oleic acid-hydroxy stearic acid; PAHPA = palmitic acid-hydroxy palmitic acid; PAHSA = palmitic acid-hydroxy stearic acid; SAHSA = stearic acid-hydroxy stearic acid

**

Supplemental Figure 1: Chemical structure of the different FAHFAs analyzed.**

FAHFA = fatty acid ester of hydroxy fatty acid; OAHSA = oleic acid-hydroxy stearic acid; PAHPA = palmitic acid-hydroxy palmitic acid; PAHSA = palmitic acid-hydroxy stearic acid; SAHSA = stearic acid-hydroxy stearic acid


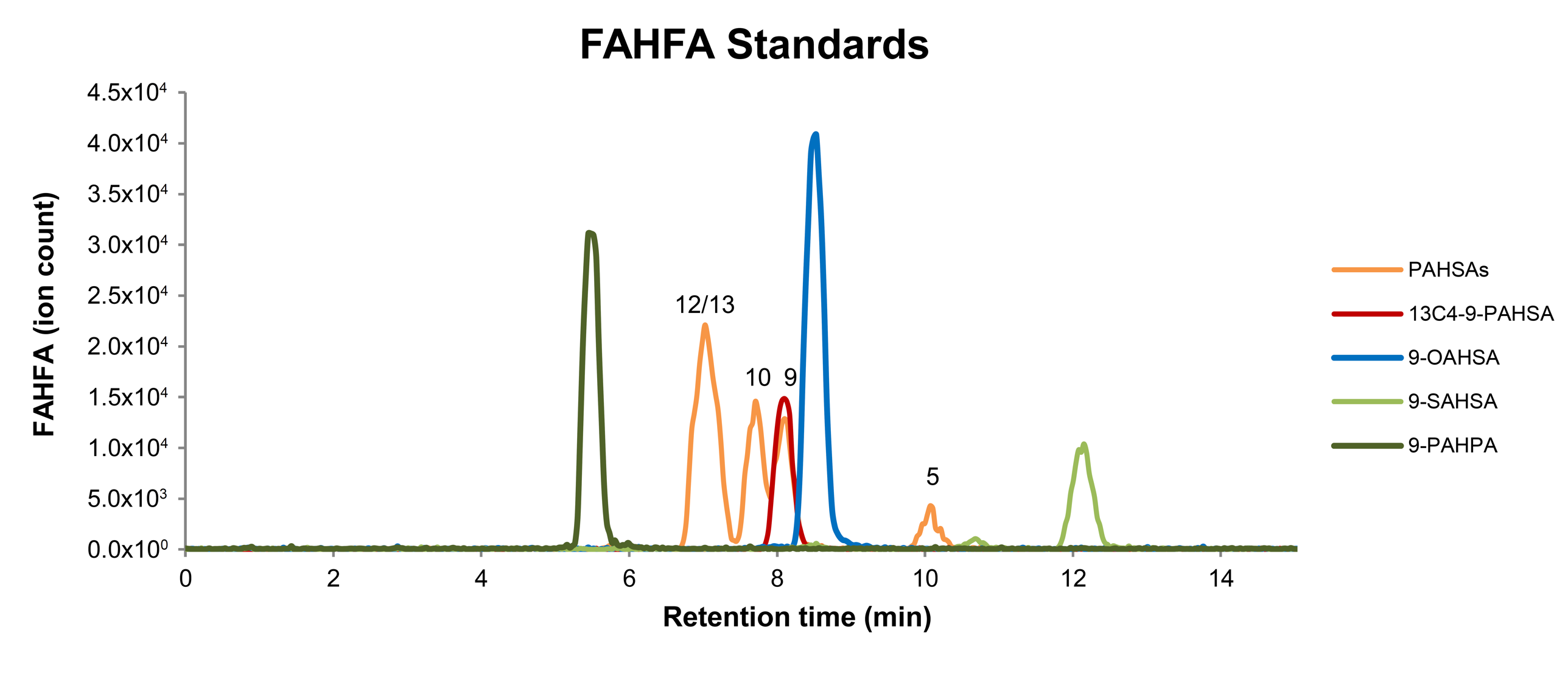
**Supplemental Figure 2: Chromatographic profile of all FAHFA standards.**

The LC-MS method enabled the separation of 5-, 9-, 10-, and 12/13-PAHSA as well as 9-OAHSA, 9-SAHSA and 9-PAHPA. ^13^C_4_-9-PAHSA was used as internal standard. The graph was created with Microsoft Excel 2010. Therefore, raw data were exported from Analyst Software.

FAHFAs = fatty acid ester of hydroxy fatty acid; OAHSA = oleic acid-hydroxy stearic acid; PAHPA = palmitic acid-hydroxy palmitic acid; PAHSA = palmitic acid-hydroxy stearic acid; SAHSA = stearic acid-hydroxy stearic acid
